# Bridging the gap in silicon photonics: quantum dot lasers and the end of the optical isolator

**DOI:** 10.1038/s41377-026-02290-w

**Published:** 2026-05-08

**Authors:** Shujie Pan, Junjie Yang, Siming Chen

**Affiliations:** 1 Laboratory of Solid State Optoelectronics Information Technology, Institute of Semiconductors, Chinese Academy of Sciences, Beijing, 100083 China; 2 HS Photonics Co., Ltd., Xiangjiang Science & Technology Innovation Base, Changsha, 413000 Hunan China; 3 College of Materials Science and Opto-Electronic Technology, University of Chinese Academy of Science, Beijing, 101804 China

**Keywords:** Semiconductor lasers, Quantum dots

## Abstract

Experiments in quantum dot lasers have demonstrated that optimized devices can withstand extreme levels of optical feedback without succumbing to coherence collapse. These results pave the way for a new generation of compact, isolator-free photonic integrated circuits.

## Abstract (web summary)

Quantum dot (QD) lasers enable intrinsically feedback-tolerant, isolator-free silicon photonic integrated circuits (PICs), eliminating the bulky optical isolators traditionally required to suppress destabilizing reflections. Owing to their delta-function-like density of states, near-zero linewidth enhancement factor, and strong damping, QD lasers sustain stable, high-speed operation without coherence collapse, even under extreme optical feedback. This intrinsic resilience enables direct monolithic integration onto silicon, paving the way for compact, scalable, and CMOS-compatible optical interconnects, while accelerating the transition from laboratory demonstrations to industrial deployment.

## Graphical abstract

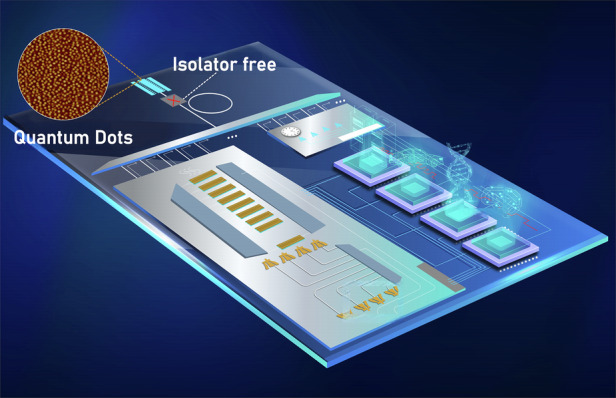

The past decades have witnessed an unprecedented surge in artificial intelligence (AI), cloud data centers, and high-performance computing (HPC), placing immense bandwidth demands on optical interconnects and accelerating the development of silicon photonics. Directly integrating high-performance lasers onto Si has represented the ultimate frontier for Si-photonics; however, the persistent challenge of optical feedback remains a critical obstacle to achieving stable monolithic integration. The parasitic reflections that originate from downstream optical components, such as grating couplers, waveguide interfaces, or fiber couplings, can re-enter the laser cavity and destabilize its operation^1^. The light interaction triggers severe phase fluctuations and noise. As these reflections reach critical feedback levels, they precipitate a transition into coherence collapse (CC), a fundamentally chaotic state that destabilizes the laser performance and renders it unsuitable for high-performance applications. To date, the standard remedy has been the inclusion of optical isolators. While effective, these components are bulky, expensive, and incompatible with the vision of fully monolithic, large-scale photonic integrated circuits (PICs). The search for a laser source inherently immune to feedback has thus become one of the field’s defining challenges.

In a recent issue of *Light: Science & Applications*^2^, Prof. Yating Wan, Prof. John Bowers, and colleagues provide compelling evidence that quantum dot (QD) lasers may offer the long-sought solution for isolator-free PICs. Owing to their discrete, delta-function-like density of states, QD-based lasers exhibits several superior characteristics, including low threshold currents^3^, high thermal stability^4–6^, low noise and long lifetime^7^. The local strain field associate on the QDs renders them insensitive to the defects^8,9^, enabling both monolithic and heterogeneous integration of QD lasers onto Si substrates, compatible with mature complementary metal oxide semiconductor (CMOS)-compatible fabrication techniques for large-scale manufacturing^10–12^. More critically, the unique density of states of QDs decouples the refractive index change from the optical gain variations, yielding a near-zero linewidth enhancement factor (${\alpha }_{H}$), the pivotal parameter governing laser’s sensitivity to optical feedback. This property, combined with the inherent ultrafast carrier dynamics in QDs, produces a high damping factor that stabilizes the laser cavity against perturbations from reflected photons and suppresses the onset of CC.

Despite the aforementioned theoretical advantages, comprehensive empirical validation has been historically hindered by experimental constraints. Previous studies were frequently limited by significant coupling losses, restricting optical feedback characterization to a threshold of –10 dB (or –13 dB when considering backward coupling loss). Recent progress includes the demonstration of error-free 128 Gbps PAM4 transmission in isolator-free packaging configurations, marking a significant step toward simplified, high-capacity silicon photonic links^13^. While QD devices appeared robust under such conditions, their ultimate tolerance limits and failure mechanisms remain unexplored. This knowledge gap has posed a significant hurdle for system-level integration, leaving open the question of whether QD lasers can maintain stability in dense PICs where reflection intensities often exceed previously accessible benchmarks (Fig. 1).

**Fig. 1:**
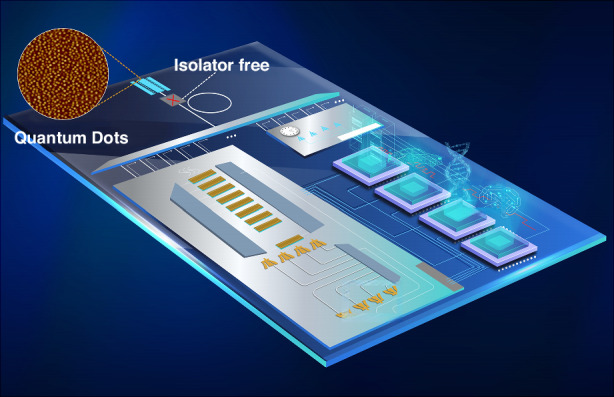
Prospects of on-chip QD lasers for PICs. Schematic illustration of isolator-free QD lasers for PICs

By leveraging a novel feedback loop integrated with a semiconductor optical amplifier, Shi et al. bypassed traditional experimental constraints to push feedback levels to a staggering 0 dB, effectively returning 100% of the emitted power into the laser cavity. Subjecting optimized InAs/GaAs QD lasers to this rigorous “torture test”, the researchers demonstrated that these devices maintain operational stability up to a threshold of –6.7 dB (~21%), a resilience that is orders of magnitude higher than that of standard quantum well lasers (which typically fail at –30 dB) and rivals complex hybrid-integrated designs. Crucially, the team validated the real-world viability of this tolerance by achieving penalty-free 10 Gbps data transmission under –7 dB feedback across a 15–45 °C thermal window, meeting a key prerequisite for uncooled datacenter applications. These findings prove that QD lasers possess an intrinsic safety margin that comfortably exceeds the cumulative reflection “noise floor” of typical PICs, effectively validating the removal of bulky optical isolators and clearing the path for truly monolithic silicon photonic integration. Meanwhile, the resilience of QDs to optical feedback is substantial evidenced by a synthesis of recent studies, as summarized in Table 1.

**Table 1:** Comparative analysis of reflection tolerance in QD Lasers

Device Type	Critical Feedback Level	Backward Coupling Loss Consideration	Transmission Results	Year/Ref
FP	−6.7 dB	Yes	10 Gbps NRZ	2025/^2^
MLL	−13 dB	Yes	128 Gbps PAM4	2025/^13^
FP	> −13 dB	-	128 Gbps PAM4	2025/^14^
MLL	> −10 dB	No	25 Gbps NRZ	2024/^15^
DFB	−14 dB	-	-	2023/^16^
FP	> −8 dB	No	10 Gbps NRZ	2020/^17^
FP	> −7.4 dB	No	25 Gbps NRZ	2019/^18^
FP	> −10 dB	-	-	2017/^19^

In conclusion, this work moves QD lasers beyond the status of a “promising” material platform and establishes them as mature, robust light sources ready for deployment in next-generation optical interconnects. By uniting fundamental material advantages with rigorous, application-driven validation, this work delivered a practical blueprint for on-chip light source for isolator-free PICs. As the demand for bandwidth, energy efficiency, and miniaturization continues to escalate, QD lasers stand poised to become the cornerstone of future silicon photonic systems — bridging the gap between laboratory innovation and industrial reality.
